# The Hertford British Hospital

**Published:** 1890-11-15

**Authors:** 


### The Hertford British Hospital.

This hospital, to judge from the many queries directed to us, seems to be but little known to the greater number of our readers. The sole institution of its kind in Paris for the succour of the 1,050 British residents, the majority of whom are poor, in the sense of being unable to pay for medical treatment, it was founded by the late Sir Richard Wallace in 1870, in memory of the Marquis of Hertford.

Like most charitable works, it had a small beginning. The first siege of Paris necessitated various ambulance organisations, and, as an appendage to one of these, Sir Richard opened a hospital in the Rue d'Aguesseau; the Commune, in the following year, found this a well-developed institution, and in the spring of 1871 two wards were added, and a dispensary opened for the relief of the British poor.

A year later the hospital was removed to Neuilly, and at that time only one foreigner remained; on his dismissal it was decided to limit the *clientèle* to British subjects, and a committee was formed of the visiting physicians and her Majesty's Consul, with the founder as Chairman.

The house in Neuilly being very unsuitable, it was decided to build a new hospital, and for this purpose a site was bought in the Rue de Villiers—Levallois Perret. Sir Richard and Lady Wallace laid the foundation stone of the present building on August 24, 1877, and in the following year it was opened under the auspices of Lord Lyons, then H.M. Ambassador in Paris.

Their Royal Highnesses the Prince and Princess of Wales visited the hospital in June of the same year, and named the large men's and women's wards the "Albert Edward" and "Alexandra," while the two smaller ones were called the "Sir Richard Wallace" and the "Lady Wallace" wards.

The plans were the production of Mons. Sanson, assisted by the Hon. Alan Herbert, M.D., and the result, as was expected from such a combination, embodied the latest scientific and architectural improvements.

The grounds are surrounded by a quartet of streets, and screened off from the public gaze by a high wall, supplemented by a belt of trees and shrubs. The massive iron gates in the Rue de Villiers give entrance to the courtyard, into which open the "big ha' door" and those of the outdoor consultation and hydropathy departments.

The structure has an ecclesiastical appearance, which is singularly appropriate, when we remember that our ancestors utilised their churches and cathedrals for the housing of the sick during times of war or pestilence; it is composed of a large central portion and two wings, which latter are mainly occupied by the large wards. The ground floor of the right wing is made up of two waiting-rooms and a consultation-room—which is also the pharmacy—for the outdoor patients; that of the left wing is the hydropathy, and contains a douché-room, vapour bath, *bains de siege*, medicated baths, a reposing, and a waiting-room. The central portion is occupied on the ground floor by a chapel, bureau, committee-room, and *chambre de concierge.* From the back of the hall lead two passages to the culinary department, which forms a small wing by itself; and another two give access to the hydropathy and out patients' rooms. The hall is about two storeys in height, and is well lighted on three sides by large ornamental windows; from it the first floor is reached by two stone staircases, one for each ward. Having mounted the right one, and passed through the glass door at its summit, we are in the passage which communicates with the bath-rooms and offices in the rear, and are admitted to the men's ward through a door on our right.

Immediately on entering we see the nurses' room on the one side, and the side ward—containing two beds—on the other; the former contains a large press for linen and various cupboards and drawers for drugs, dressings, and other necessaries. A fireplace, gas stove, and sink are included in the furnishings, though the nurse does neither cooking nor washing up. Passing on to the ward one is astonished at the height and dome-shaped appearance of the roof, giving an amount of space which is invaluable in the treatment of chest cases, and particularly of consumption. The grating down the centre of the ward next arrests attention; under it are two pipes, one containing hot air and the other steam for heating purposes. There are twelve beds in the ward, including the two cribs, and these, with the side-room ones, make up a total of fourteen. The far end of the ward is round, and contains a couch, fire-place, and a table. This little recess, whose roof is about half the height of the ward, can accommodate eight seats round the table, and from it exit is given by means of a glass door, which opens to the front, and a balcony-staircase, which curves round the end of the house and descends by a gentle slope to the garden.

Ventilation is amply provided for by the large Ogival windows, which can be opened in sections by means of a key, aided by small traps communicating with the exterior near the floor, and also by the tunnel system, in which an upward current is produced for the exit of foul air by lighting a Bunsen burner in each tunnel.

The female ward is identical to the one described, from which it is separated by a double partition; between these two partitions the staircase from the first to the second stage is placed, and behind this the lift is situated.

Mounting to the second stage we see the two small wards, containing three beds each, and separated by the nurses' room. They are occupied by convalescent patients, have each a balcony, which is a favourite resort in fine weather. On this floor are the lady superintendent's and house surgeon's rooms, so situated that they have windows looking into the two female and the two male wards respectively; and here, too, is the operation room. The nurses' and servants' rooms are on the third floor, as are also the linen and blanket rooms; the latter are very extensive from the fact that the hospital supplies every article of raiment for each patient during his residence in the wards, necessitating a large stock. In the attics are the various store-rooms for all kinds of dry goods, beds and bedding, splints and appliances; and here, too, is the large electric clock, from which proceed wires to the various dials in the house.

Descending to the basement, we see the two long gardens separated by a gravel path, which is flanked by an iron railing, overgrown with ivy; at the foot of this pathway are two buildings, one containing a mortuary and an autopsy room, the other a chapel for burial services, and a sorting room for soiled linen. These all open into a courtyard which communicates with the new chapel, so that all funeral processions are out of sight of the patients.

The hospital was endowed by its founder, and is independent of all external aid. The management consists of a committee which controls financial matters, and generally superintends the working of the establishment. The lady superintendent and her assistant have for their especial charge the nursing and household departments, while the house surgeon is responsible for the treatment of the patients and the medical and surgical stores.

The nursing staff consists of five trained nurses, and one probationer; the four who are attached to the large wards take night duty in rotation every two months, and change from male to female side, and *vice versâ*, about three times a year.

The nurse of the convalescent wards has no night work, and her hours of duty are from seven a.m. till eight p.m.

The probationer is on duty at seven in the morning, an hour before the day nurses, and she is off duty two hours earlier at eight p.m.

The salary is £28 a-year, with travelling expenses, and the work, though not very heavy, is both medical and surgical. Changes in the staff are not very frequent, and the list of applicants is always large. The nurses have opportunity for learning or perfecting their French, and for seeing a variety of cases and forms of treatment, which go to make up "experience." The drawback is that nurses require to study economy and practice it in order to make their salary include uniform and last over the year; for money does not go so far in Paris as in England.

The patients are of a better class than we generally have in an English hospital, and so more tact is required for their management. No infectious cases are admitted save typhoid fever, and it, with ordinary precautions, is quite safe. Chest diseases, notably phthisis pulmonalis, are a constant item in the diagnosis book of the male side. Amongst the women the most common affection is anæmia and general exhaustion (usually in governesses and servants), typhoid fever, and gynæcology, with a variety of ordinary medical and surgical work. In the twenty years of its existence the hospital has been of immense value to the British poor in Paris, and is an example which America's millionaires might follow for the relief of their countrymen who are not eligible for the Hertford British Hospital.

